# Effects of high-intensity interval training on cardiopulmonary function and quality of life in patients with myocardial infarction: a meta-analysis

**DOI:** 10.3389/fcvm.2025.1666325

**Published:** 2025-12-15

**Authors:** Xiening Xu, Ying Shen, Yuying Lao

**Affiliations:** Department of Emergency, Shaoxing Second Hospital, Shaoxing, China

**Keywords:** high-intensity interval training, myocardial infarction, peak oxygen uptake, respiratory exchange ratio, quality of life, meta-analysis

## Abstract

**Background:**

High-intensity interval training (HIIT) is increasingly used in cardiac rehabilitation. Its effects on cardiopulmonary function and quality of life in patients with myocardial infarction (MI) require systematic evaluation.

**Methods:**

A systematic search was conducted in PubMed, Embase, Web of Science, Cochrane Central, and CNKI from inception to April 1, 2025. Randomized controlled trials assessing the effects of HIIT on peak heart rate (HRpeak), peak oxygen uptake (VO₂peak), respiratory exchange ratio (RER), and quality of life (QOL) in post-MI patients were included. Data were synthesized using a random-effects model. Standardized mean differences (SMDs) and 95% confidence intervals (CIs) were calculated. Heterogeneity was assessed with the I² statistic, and sensitivity analyses were performed.

**Results:**

Nine trials with 508 participants were included. HIIT did not significantly affect HRpeak (SMD = −0.20; 95% CI: −1.10 to 0.70; I^2^ = 62.4%) or VO₂peak (SMD = 0.32; 95% CI: −0.07 to 0.71; I^2^ = 69.1%). RER was significantly reduced in the HIIT group (SMD = −1.26; 95% CI: −1.54 to −0.98; I^2^ = 0%). No significant improvement was observed in QOL (SMD = 0.07; 95% CI: −0.37 to 0.50; I^2^ = 0%). Sensitivity analyses suggested that individual studies contributed to heterogeneity in HRpeak and VO₂peak outcomes.

**Conclusions:**

HIIT may influence selected cardiopulmonary indicators in post-MI patients, particularly metabolic efficiency. Its effects on exercise capacity and quality of life remain uncertain. Further research with standardized protocols is needed.

## Introduction

Myocardial infarction (MI) remains one of the leading causes of morbidity and mortality worldwide, posing a substantial burden on healthcare systems and adversely affecting patients' functional capacity and quality of life (1–3). Despite advancements in reperfusion therapies, pharmacological interventions, and secondary prevention strategies, a significant proportion of post-MI patients continue to experience reduced cardiopulmonary function, persistent symptoms, and impaired psychosocial well-being (4, 5). These limitations not only diminish patients' ability to resume daily activities but are also closely associated with long-term prognosis and cardiovascular event recurrence (6, 7).

Cardiac rehabilitation (CR) has been firmly established as a cornerstone of secondary prevention in patients with coronary artery disease, with robust evidence supporting its role in improving exercise tolerance, modulating cardiovascular risk factors, and reducing mortality. Traditionally, moderate-intensity continuous training (MICT) has been the predominant exercise modality employed in CR programs. However, recent years have witnessed growing interest in high-intensity interval training (HIIT), an alternative exercise paradigm characterized by brief bursts of vigorous activity interspersed with periods of active recovery (8). HIIT has been proposed to elicit superior physiological adaptations through greater mechanical and metabolic stimulus, potentially leading to more pronounced improvements in maximal oxygen uptake (VO₂peak), endothelial function, and autonomic regulation (9, 10).

Emerging clinical studies suggest that HIIT may confer greater cardiopulmonary benefits than MICT in post-MI populations, with improvements in functional capacity translating into enhanced health-related quality of life (HRQoL) (11, 12). Moreover, the time efficiency and motivational appeal of HIIT may improve adherence among patients who are often reluctant to engage in prolonged exercise sessions (13). Nonetheless, findings across randomized trials remain heterogeneous, and concerns regarding the safety, feasibility, and generalizability of HIIT in high-risk cardiac populations persist. Given these uncertainties, there is a critical need to systematically synthesize available evidence to clarify the clinical value of HIIT in this context.

This meta-analysis aims to comprehensively evaluate the effects of high-intensity interval training on cardiopulmonary function and quality of life in patients recovering from myocardial infarction. By integrating data from randomized controlled trials and assessing the magnitude, consistency, and clinical significance of outcomes, this study seeks to inform evidence-based exercise prescription and guide future research directions in cardiac rehabilitation.

## Methods

### Search strategy

A systematic and comprehensive search of the literature was undertaken in five major databases: PubMed, Embase, Web of Science, Cochrane Library, and China National Knowledge Infrastructure (CNKI), from inception to April 1, 2025. The search strategy combined both Medical Subject Headings (MeSH) and free-text terms related to myocardial infarction, high-intensity interval training, aerobic capacity, cardiopulmonary rehabilitation, and quality of life. Boolean operators (“AND,” “OR”) were applied to combine terms appropriately. The search strategy was peer-reviewed and tailored to each database. No language restriction was applied during the search. A complete list of search terms and strategies is provided in Supplementary Table S1. Reference lists of included studies and relevant reviews were also manually screened to ensure comprehensiveness.

### Inclusion and exclusion criteria

Studies were eligible for inclusion if they met the following criteria: (1) Randomized controlled trials (RCTs) comparing HIIT with usual care. (2) Adult patients (≥18 years) with a confirmed diagnosis of MI (by clinical, electrocardiographic, or biomarker criteria), regardless of sex, ethnicity, or infarct location. (3) At least one of the following was reported post-intervention: peak oxygen uptake (VO₂peak), peak heart rate (HR peak), peak respiratory exchange ratio (peak RER) or validated scales of health-related quality of life (14).

Studies were excluded if they met any of the following criteria: (1) Non-randomized, observational, or quasi-experimental designs; (2) Duplicate publications from the same cohort without additional information; (3) Conference abstracts, reviews, editorials, or animal studies.

### Data extraction and management

Two reviewers independently extracted data from each eligible study using a standardized form. Extracted information included basic study characteristics such as author, year of publication, sample size, and participant demographics, as well as outcome indicators. When essential data were not directly reported, efforts were made to contact study authors for clarification. Any discrepancies in the extraction process were resolved through consensus or consultation with a third reviewer.

### Risk of bias assessment

The risk of bias in each included study was evaluated independently by two reviewers using the Cochrane Risk of Bias 2.0 tool, which assesses five key domains: (1) bias arising from the randomization process, (2) deviations from intended interventions, (3) missing outcome data, (4) measurement of the outcome, and (5) selection of the reported result. Each domain was rated as “low risk,” “some concerns,” or “high risk.” An overall risk of bias judgment was generated for each study. Discrepancies were resolved by consensus or third-party arbitration.

### Statistical analysis

All meta-analyses were conducted using R (version 4.3.3). For continuous variables, standardized mean differences with corresponding 95% confidence intervals were calculated. When analyzing dichotomous outcomes, risk ratios with 95% confidence intervals were used. Given the anticipated variability in study populations and intervention protocols, a random-effects model was applied. Statistical heterogeneity was evaluated using the I² statistic, with values of 25%, 50%, and 75% considered indicative of low, moderate, and high heterogeneity, respectively. To examine the robustness of the findings, sensitivity analyses were performed by sequentially excluding studies with methodological limitations or small sample sizes.

## Result

### Study selection and characteristics

**Figure 1:**
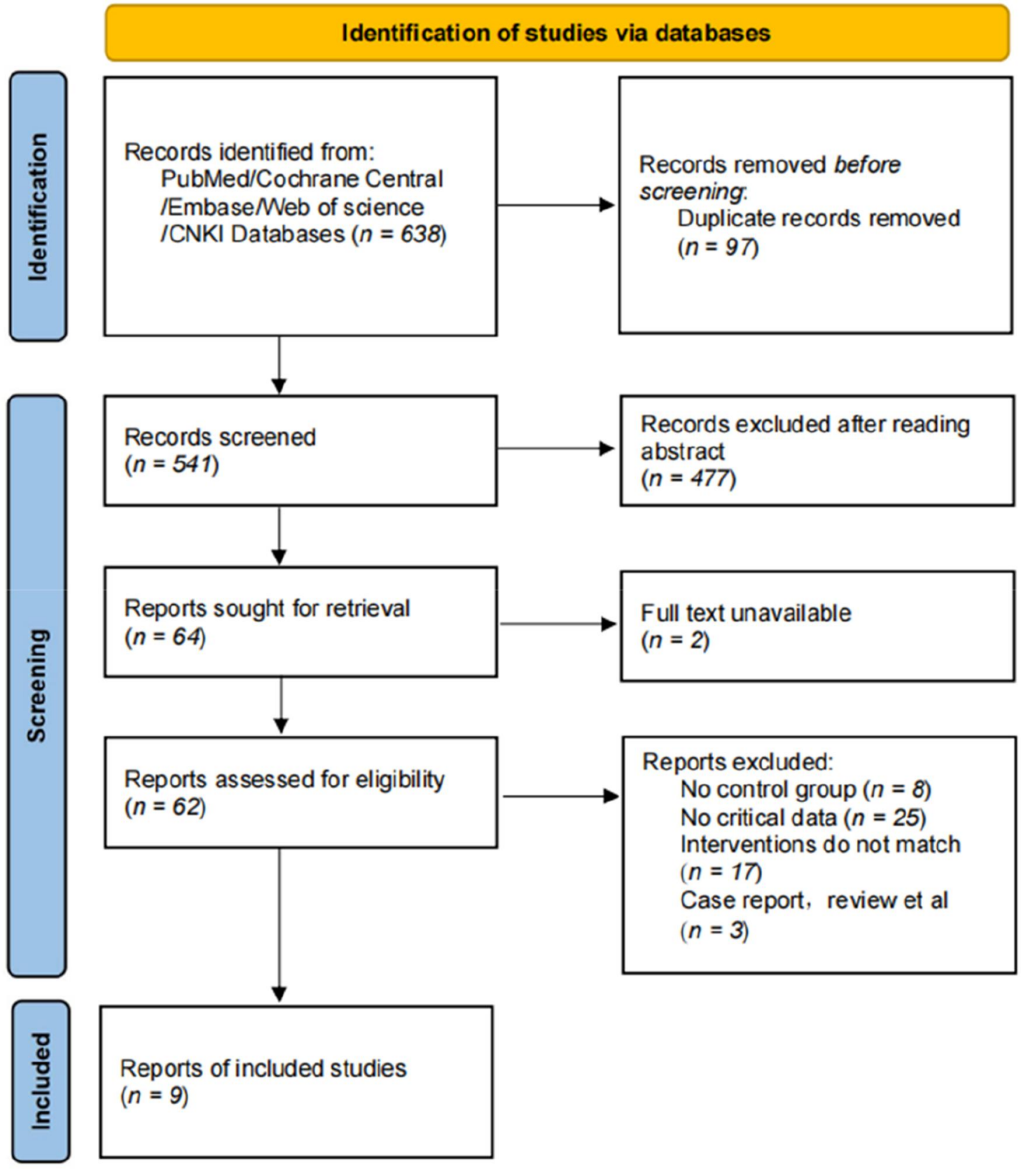
PRISMA flow diagram.

A total of 638 articles were initially retrieved from PubMed, Cochrane Central, Embase, Web of Science, and CNKI. After removing duplicates and screening titles, abstracts, and full texts according to predefined criteria, nine randomized controlled trials (15–23) were ultimately included in the meta-analysis. The selection process is outlined in the PRISMA flow diagram (Figure 1), and the key characteristics of the included studies are summarized in Table 1.

**Table 1:** Baseline characteristics.

First author	Publication year	Number of cases		Age (year)		Gender	
						(Male/Female)	
		Experimental group	Control group	Experimental group	Control group	Experimental group	Control group
Eser et al. (15)	2022	34	35	53 (49–66)	59 (52–62)	/	/
JonAnder et al. (16)	2022	28	14	/	/	23/5	12/2
Marcin et al. (17)	2022	35	34	55 (50–66)	59 (50–62)	/	/
Moholdt et al. (18)	2012	30	59	56.7 ± 10.4	57.7 ± 9.3	25/5	49/10
Ji et al. (19)	2017	28	40	50.9 ± 6.6	50.8 ± 6.5	61/35	26/14
Nam et al. (20)	2024	29	32	58.69 ± 12.38	56.66 ± 9.50	25/4	28/4
Trachsel et al. (21)	2019	9	10	60 ± 10	57 ± 13	6/3	7/3
Yakut et al. (22)	2022	11	10	59.6 ± 4.5	58.5 ± 5.6	10/1	8/2
Yi et al. (23)	2021	39	31	55.63 ± 6.37	56.38 ± 7.06	21/18	18/13

### Risk of bias assessment

**Figure 2:**
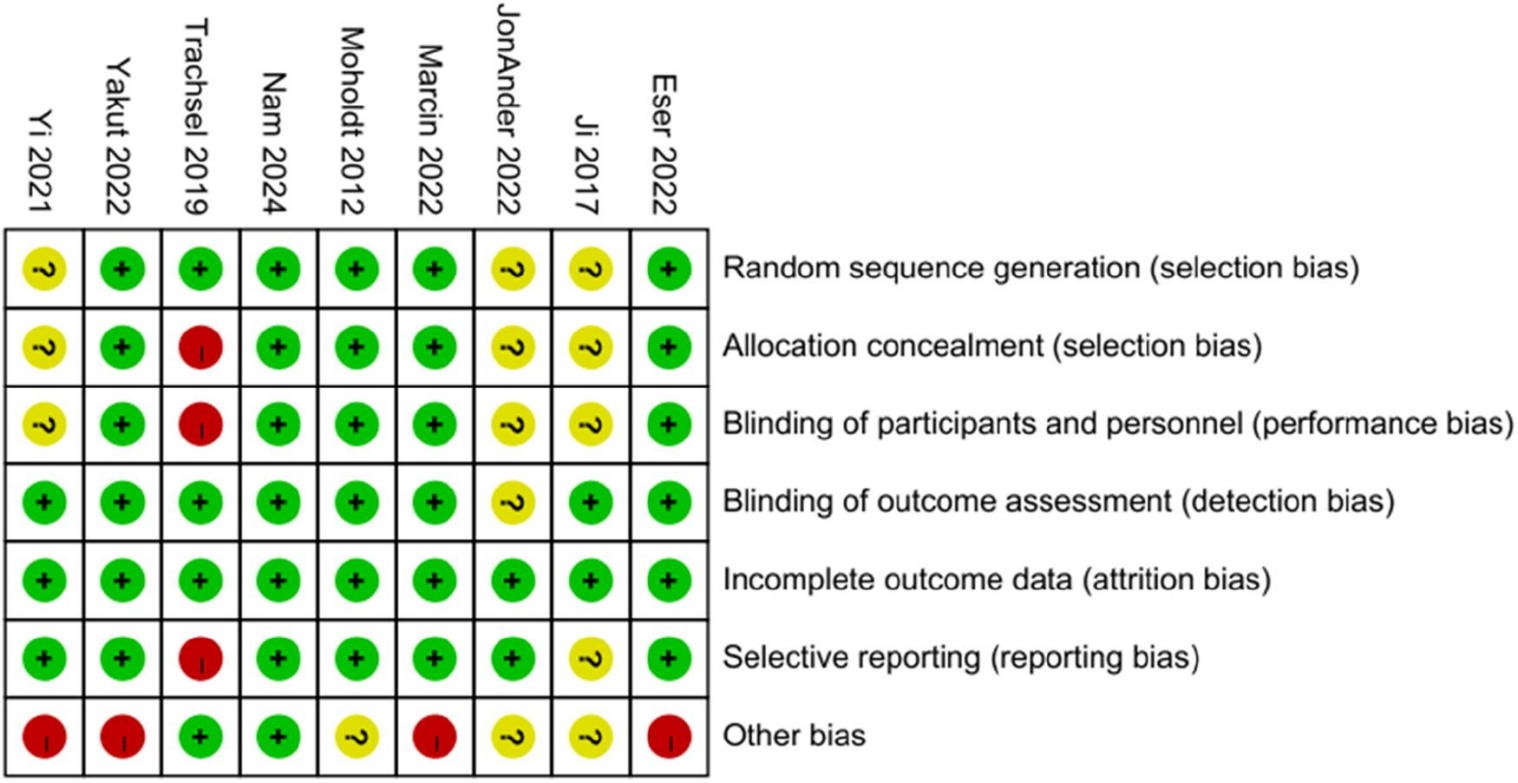
Risk of bias summary.

**Figure 3:**
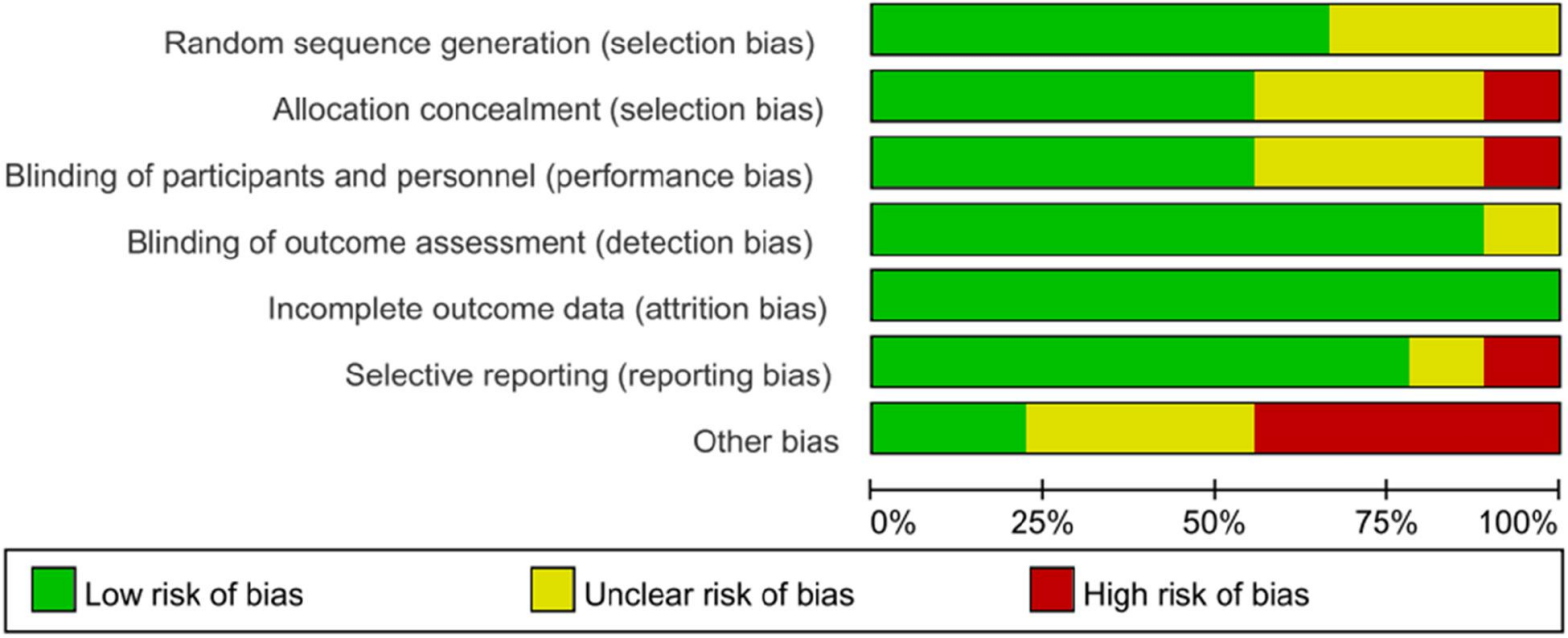
Risk of bias graph.

The 9 included studies were all RCTs. Most of these studies demonstrated high methodological quality, particularly in terms of random sequence generation, blinding, allocation concealment, and completeness of outcome reporting. However, a few studies showed uncertainty regarding allocation concealment (Figures 2, 3).

### Effect of HIIT on peak heart rate

**Figure 4:**
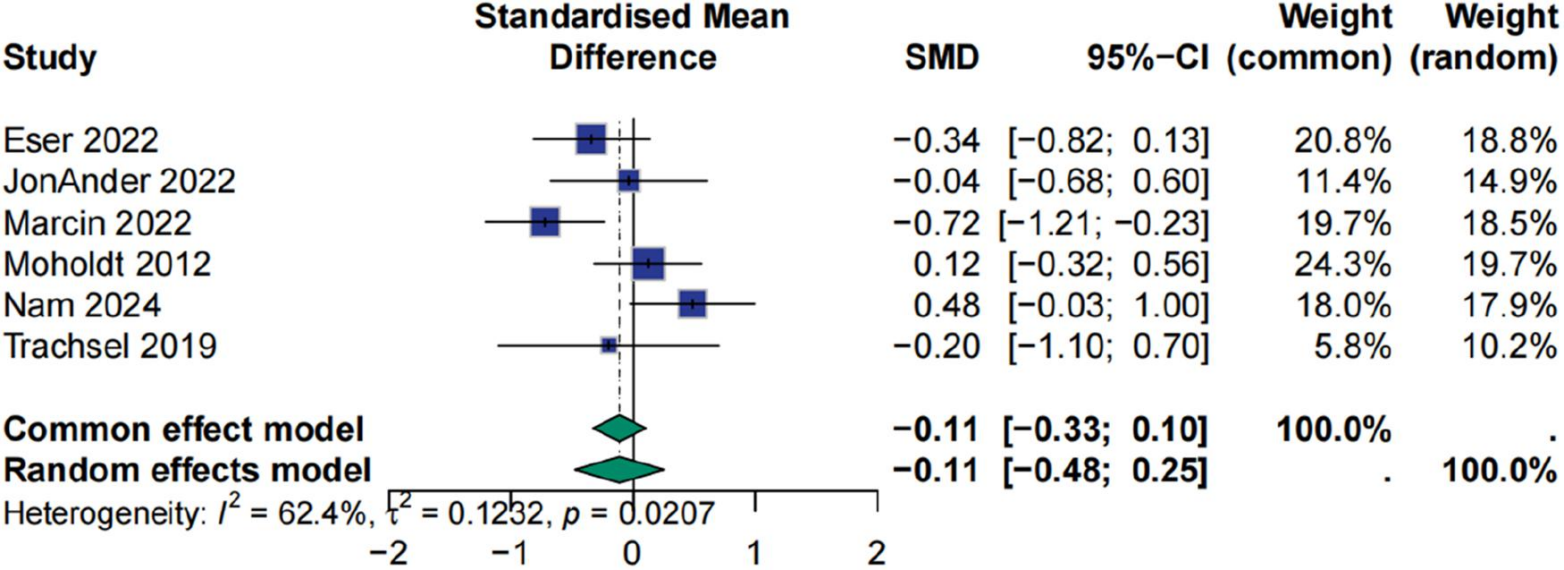
Meta analysis of peak heart rate.

**Figure 5:**
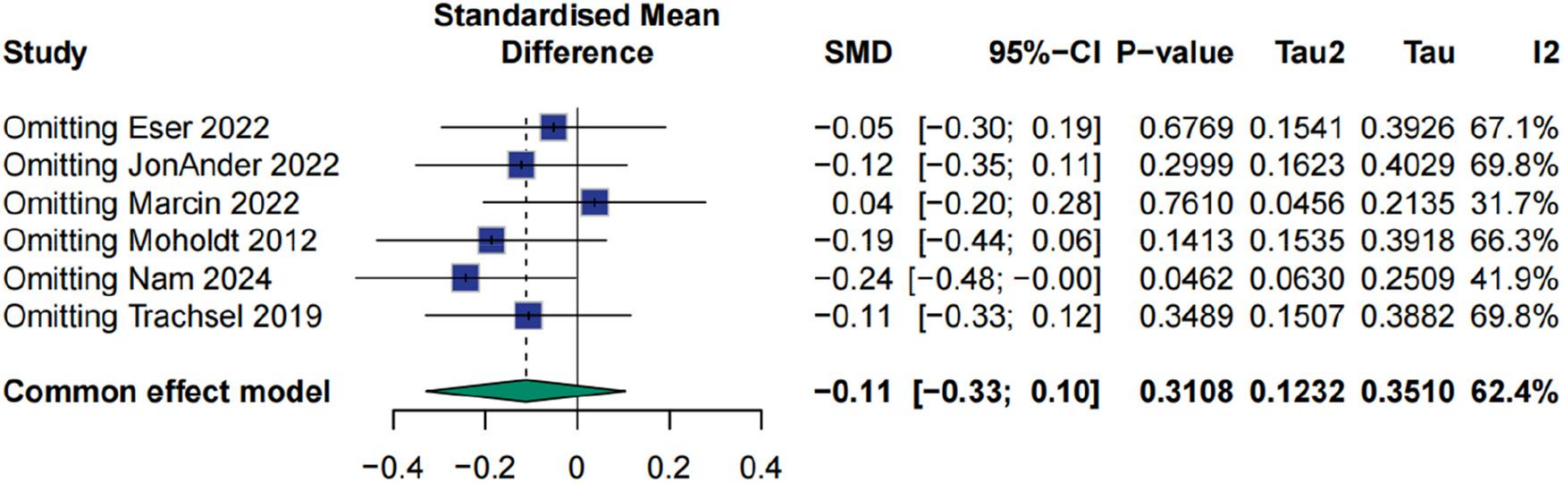
Sensitive analysis of peak heart rate.

The impact of high-intensity interval training (HIIT) on peak heart rate in patients with myocardial infarction was evaluated across six studies. The pooled analysis using a random-effects model indicated that HIIT did not significantly improve peak heart rate compared with conventional interventions, with a standardized mean difference of −0.11 and a 95% confidence interval from −0.48 to 0.25 (*p* > 0.05). Moderate heterogeneity was observed across studies (I² = 62.4%), potentially attributable to variations in training intensity, frequency, and duration. Sensitivity analysis revealed that exclusion of the study by Marcin et al. (2022) substantially reduced heterogeneity to 31.7%, although the direction and magnitude of the effect remained stable (SMD = 0.04, 95% CI: −0.20 to 0.28, *p* > 0.05) (Figures 4, 5).

### Effect of HIIT on peak oxygen uptake

**Figure 6:**
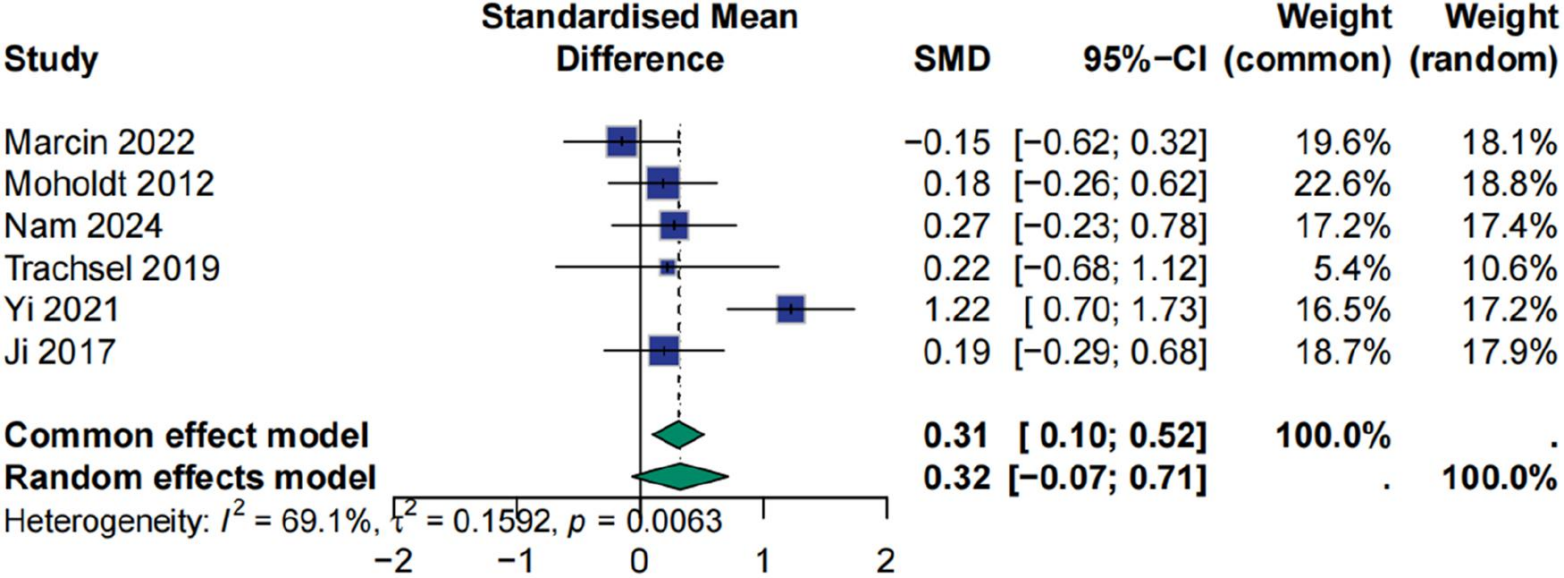
Meta analysis of peak oxygen uptake.

**Figure 7:**
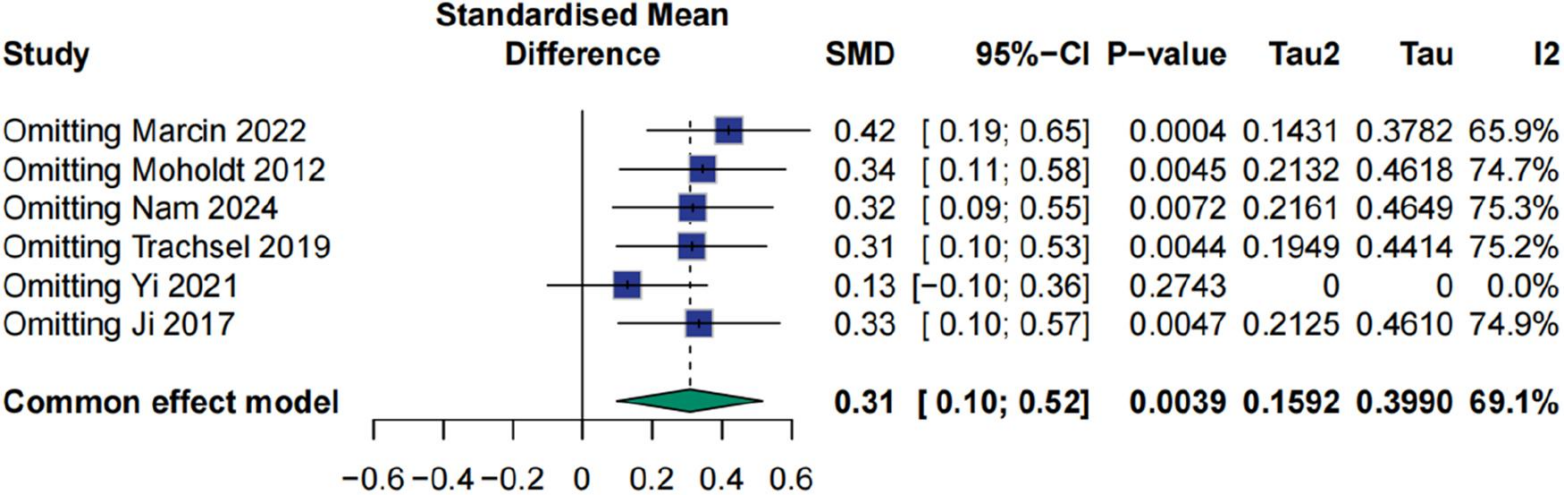
Sensitive analysis of peak oxygen uptake.

Analysis of Peak VO₂, a key marker of aerobic capacity, showed a positive trend associated with HIIT. The pooled effect size was 0.32 (95% CI: −0.07 to 0.71), *p* > 0.05, indicating that the result did not meet the conventional threshold for statistical significance. Substantial heterogeneity was detected (I² = 69.1%). Sensitivity analysis identified the study by Yi et al. (2021) as a major source of heterogeneity; exclusion of this study reduced heterogeneity to 0%, but concurrently decreased the effect size to 0.13 (95% CI: −0.10 to 0.36, *p* = 0.2743) (Figures 6, 7).

### Effect of HIIT on respiratory exchange ratio

**Figure 8:**
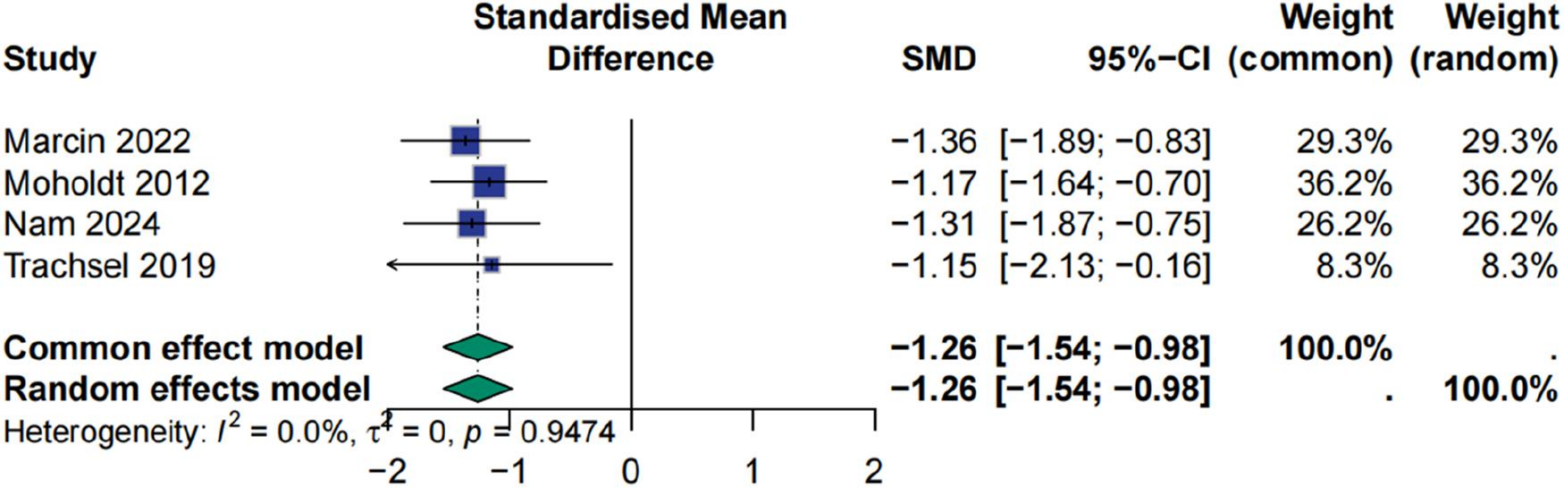
Meta analysis of respiratory exchange ratio.

**Figure 9:**
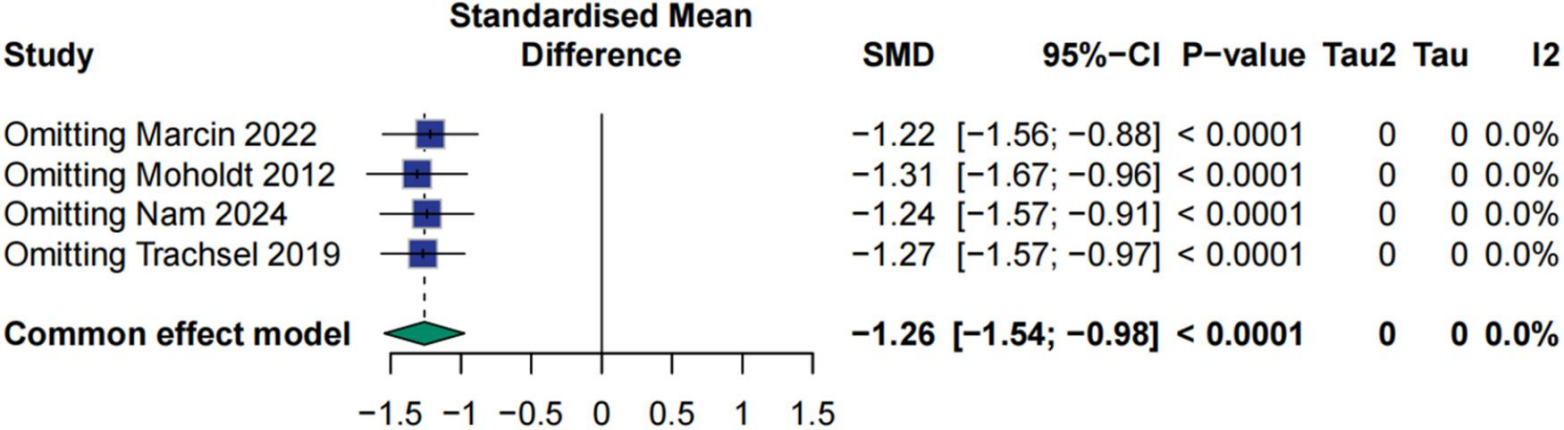
Sensitive analysis of respiratory exchange ratio.

The RER showed a clear and consistent improvement following HIIT. The meta-analysis demonstrated a significant reduction in RER among patients undergoing HIIT compared to controls, with a standardized mean difference of −1.26 (95% CI: −1.54 to −0.98, *p* < 0.0001). No heterogeneity was observed across studies (I² = 0%), and sensitivity analyses confirmed the robustness of the findings, with minimal variation in effect size (±0.05) upon exclusion of individual studies (Figures 8, 9).

### Effect of HIIT on quality of life

**Figure 10:**
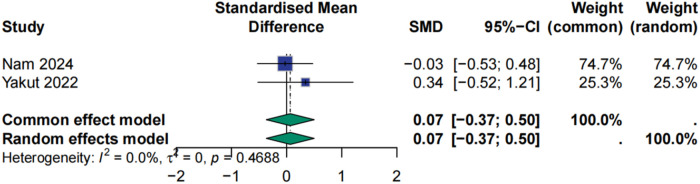
Meta analysis of quality of life.

Evidence regarding the impact of HIIT on QOL remains limited. Only two studies met inclusion criteria for this outcome. The combined effect was not statistically significant (SMD = 0.07, 95% CI: −0.37 to 0.50, *p* = 0.7562), and no heterogeneity was detected (I² = 0%). Closer examination revealed that the study by Nam et al. (2024) reported a neutral effect, while Yakut et al. (2022) demonstrated a slight but non-significant negative trend. Although these findings suggest that HIIT may have a minimal impact on QOL in this population, the limited number of trials and variability in assessment tools underscore the need for future studies employing standardized instruments and longer follow-up durations to clarify this relationship (Figure 10).

## Discussion

This meta-analysis investigated the impact of HIIT on cardiopulmonary function and quality of life in patients following MI. The findings suggest that HIIT may induce favorable adaptations in respiratory metabolism, while evidence for its effects on heart rate dynamics and subjective quality of life remains limited and heterogeneous (24, 25). These variations likely reflect differences in the physiological mechanisms underlying each outcome, as well as the diversity in intervention protocols, patient characteristics, and assessment methods across studies (26).

The consistent improvement in RER observed in this analysis indicates enhanced metabolic flexibility following HIIT (27). RER reflects the balance between carbohydrate and fat oxidation during exercise, and its reduction suggests a shift toward greater reliance on fat metabolism (28). In the context of MI, where patients often exhibit impaired mitochondrial function and reduced oxidative capacity, HIIT may stimulate mitochondrial biogenesis and improve efficiency through repeated bouts of high-intensity stress, thereby restoring energy homeostasis (29). This metabolic shift is clinically meaningful, as it may enhance myocardial energy supply and reduce the burden on the ischemic myocardium during physical activity.

While improvements in RER were robust, changes in VO₂peak were more variable. VO₂peak is a multifactorial parameter influenced by central cardiac output, peripheral oxygen extraction, and muscular oxidative capacity (30). Although HIIT has the potential to improve all these components, the extent of adaptation depends on the training volume, intensity, and duration. Studies included in this meta-analysis differed markedly in terms of exercise intensity prescription, interval structure, and total training load, and the type of exercise ergometer used, which likely contributed to the observed heterogeneity (31). In addition, evidence from patients with heart failure and reduced ejection fraction suggests that patient characteristics may further influence VO_2_ peak responses, as highlighted in a recent systematic review and meta-analysis (32, 33). VO₂ at ventilatory threshold (VO₂-VT1) represents a valuable complementary parameter, as it reflects submaximal aerobic efficiency, integrates early metabolic and ventilatory adaptations, and is less influenced by patient motivation or the ability to reach true maximal effort (34). VO₂-VT1 may therefore offer a more stable and physiologically informative indicator of training-induced improvements, particularly in populations with cardiovascular limitations.

In contrast, the effects of HIIT on HR peak appeared inconsistent. Peak heart rate reflects autonomic nervous system regulation and chronotropic competence, both of which are frequently altered in post-MI patients. The widespread use of beta-blockers and other rate-limiting medications likely blunted the responsiveness of HRpeak to training. Moreover, short-duration interventions may not provide sufficient stimulus to improve autonomic balance or vagal tone. These pharmacological and physiological constraints may explain the limited observed benefit in this parameter, despite trends toward improvement in several studies (35).

The evidence regarding quality of life remains inconclusive, primarily due to the limited number of trials addressing this outcome. Quality of life is a complex construct influenced by physical, psychological, and social domains. Notably, improvements in cardiorespiratory endurance have been shown to exert a direct and meaningful impact on quality of life by enhancing functional capacity, reducing fatigue, and promoting psychological well-being (36). However, such physiological benefits may not be immediately reflected in subjective perceptions of well-being, especially in the early stages of recovery when psychological distress and uncertainty about prognosis are common (29). Therefore, future studies should adopt standardized and longitudinal assessments to better elucidate the causal and dynamic relationship between cardiorespiratory endurance and health-related quality of life.

Mechanistically, HIIT may exert its benefits through multiple pathways. On a cellular level, it activates key metabolic regulators such as AMP-activated protein kinase (AMPK), enhancing fatty acid oxidation and mitochondrial efficiency. Vascular adaptations, driven by intermittent shear stress during high-intensity bouts, may improve endothelial function and peripheral perfusion (22). Moreover, HIIT has been associated with favorable modulation of autonomic tone and systemic inflammation, both of which are implicated in post-MI recovery (37). These integrated physiological responses may collectively contribute to improved functional capacity and, potentially, long-term cardiovascular outcomes.

This meta-analysis has several strengths, including a systematic literature search, adherence to PRISMA guidelines, the use of robust random-effects models, and a specific focus on peak RER as a key metabolic outcome in post-MI patients undergoing HIIT. However, the findings must be interpreted in the context of several limitations. First, the overall number of included studies was relatively small, which may limit the generalizability of the findings and the statistical power for some outcomes. Second, we observed moderate-to-high heterogeneity in the analyses of HRpeak and VO_2_peak, which, despite our sensitivity analyses, could not be fully resolved. This is likely attributable to the variability in HIIT protocols and patient characteristics across studies. Third, as noted earlier, the assessment of QOL was limited by the small number of studies and the use of different measurement tools. Fourth, although VO_2_ at ventilatory threshold (VO_2_-VT₁) and post-training peak RER are potentially informative indicators of submaximal functional capacity and exercise-induced adaptations, limited reporting and inconsistency across studies prevented us from performing subgroup analyses or meta-regression to formally evaluate these effect modifiers. Future meta-analyses with a larger pool of primary studies and more comprehensive reporting are warranted to investigate these effect modifiers. Future meta-analyses with a larger pool of primary studies are warranted to investigate these effect modifiers.

In conclusion, this meta-analysis suggests that HIIT can significantly improve peak respiratory exchange ratio in patients recovering from myocardial infarction, indicating a potential positive effect on metabolic efficiency. However, its effects on peak heart rate, peak oxygen uptake, and quality of life remain inconclusive, partly due to heterogeneity and limited data. Future high-quality RCTs with standardized HIIT protocols, consistent outcome assessments, and longer follow-up durations are needed to definitively establish the role of HIIT in improving cardiopulmonary function and overall well-being in this patient population.

## Data Availability

The original contributions presented in the study are included in the article/Supplementary Material, further inquiries can be directed to the corresponding author.

## References

[1] Johny E Dutta P. Targeting extramedullary hematopoiesis and cardiac immune homing to encourage cardiac healing after myocardial infarction. Circulation. (2025) 151(24):1748–51. 10.1161/circulationaha.125.074786 40523052 PMC12173427

[2] Sammour YM Khan SU Hong H Wu J Fanaroff AC Reed GW Institutional variability in processes of care and outcomes among patients with STEMI in the US. JAMA Cardiol. (2025) 10(8):787–96. 10.1001/jamacardio.2025.1411 40498491 PMC12159851

[3] Vogel B Claessen BE Arnold SV Chan D Cohen DJ Giannitsis E ST-segment elevation myocardial infarction. Nat Rev Dis Primers. (2019) 5(1):39. 10.1038/s41572-019-0090-3 31171787

[4] Liu F Wanigatunga AA Zampino M Knuth ND Simonsick EM Schrack JA Association of mitochondrial function, substrate utilization, and anaerobic metabolism with age-related perceived fatigability. J Gerontol A Biol Sci Med Sci. (2021) 76(3):426–33. 10.1093/gerona/glaa201 32803242 PMC8355455

[5] Van Hooren B Souren T Bongers BC. Accuracy of respiratory gas variables, substrate, and energy use from 15 CPET systems during simulated and human exercise. Scand J Med Sci Sports. (2024) 34(1):e14490. 10.1111/sms.14490 37697640

[6] Franklin BA Thompson PD Al-Zaiti SS Albert CM Hivert MF Levine BD Exercise-related acute cardiovascular events and potential deleterious adaptations following long-term exercise training: placing the risks into perspective-an update: a scientific statement from the American Heart Association. Circulation. (2020) 141(13):e705–36. 10.1161/cir.0000000000000749 32100573

[7] Rognmo Ø Moholdt T Bakken H Hole T Mølstad P Myhr NE Cardiovascular risk of high- versus moderate-intensity aerobic exercise in coronary heart disease patients. Circulation. (2012) 126(12):1436–40. 10.1161/circulationaha.112.123117 22879367

[8] Eser P Trachsel LD Marcin T Herzig D Freiburghaus I De Marchi S Short- and long-term effects of high-intensity interval training vs. moderate-intensity continuous training on left ventricular remodeling in patients early after ST-segment elevation myocardial infarction-the HIIT-EARLY randomized controlled trial. Front Cardiovasc Med. (2022) 9:869501. 10.3389/fcvm.2022.869501 35783836 PMC9247394

[9] Taylor JL Holland DJ Keating SE Leveritt MD Gomersall SR Rowlands AV Short-term and long-term feasibility, safety, and efficacy of high-intensity interval training in cardiac rehabilitation: the FITR heart study randomized clinical trial. JAMA Cardiol. (2020) 5(12):1382–9. 10.1001/jamacardio.2020.3511 32876655 PMC7489382

[10] McGregor G Powell R Begg B Birkett ST Nichols S Ennis S High-intensity interval training in cardiac rehabilitation: a multi-centre randomized controlled trial. Eur J Prev Cardiol. (2023) 30(9):745–55. 10.1093/eurjpc/zwad039 36753063

[11] McGregor G Nichols S Hamborg T Bryning L Tudor-Edwards R Markland D High-intensity interval training versus moderate-intensity steady-state training in UK cardiac rehabilitation programmes (HIIT or MISS UK): study protocol for a multicentre randomised controlled trial and economic evaluation. BMJ Open. (2016) 6(11):e012843. 10.1136/bmjopen-2016-012843 27852718 PMC5129054

[12] Ingle L Powell R Begg B Birkett ST Nichols S Ennis S Effects of exercise training response on quality of life and cardiovascular risk factor profiles in people with coronary artery disease: insights from the HIIT or MISS UK trial. Arch Phys Med Rehabil. (2024) 105(8):1464–70. 10.1016/j.apmr.2024.03.002 38493909

[13] Quindry JC Franklin BA Chapman M Humphrey R Mathis S. Benefits and risks of high-intensity interval training in patients with coronary artery disease. Am J Cardiol. (2019) 123(8):1370–7. 10.1016/j.amjcard.2019.01.008 30732854

[14] Wang W Lopez V Ying CS Thompson DR. The psychometric properties of the Chinese version of the SF-36 health survey in patients with myocardial infarction in mainland China. Qual Life Res. (2006) 15(9):1525–31. 10.1007/s11136-006-0012-1 16826443

[15] Eser P Jaeger E Marcin T Herzig D Trachsel LD Wilhelm M. Acute and chronic effects of high-intensity interval and moderate-intensity continuous exercise on heart rate and its variability after recent myocardial infarction: a randomized controlled trial. Ann Phys Rehabil Med. (2022) 65(1):101444. 10.1016/j.rehab.2020.09.008 33091614

[16] Ander J Jurio-Iriarte B Aispuru GR Villar-Zabala B Blanco-Guzman S Maldonado-Martín S. Chronotropic responses to exercise and recovery in myocardial infarction patients taking β-blockers following aerobic high-intensity interval training. J Cardiopulm Rehabil Prev. (2022) 42(1):22–7. 10.1097/hcr.0000000000000607 34793361

[17] Marcin T Trachsel LD Dysli M Schmid JP Eser P Wilhelm M. Effect of self-tailored high-intensity interval training versus moderate-intensity continuous exercise on cardiorespiratory fitness after myocardial infarction: a randomised controlled trial. Ann Phys Rehabil Med. (2022) 65(1):101490. 10.1016/j.rehab.2021.101490 33450366

[18] Moholdt T Aamot IL Granøien I Gjerde L Myklebust G Walderhaug L Aerobic interval training increases peak oxygen uptake more than usual care exercise training in myocardial infarction patients: a randomized controlled study. Clin Rehabil. (2011) 26(1):33–44. 10.1177/0269215511405229 21937520

[19] yanqiong J Chunyan Z. The impact of aerobic rehabilitation exercise intensity on myocardial perfusion, cardiopulmonary function, and quality of life in patients with myocardial infarction. Pract J Cardiocerebrovasc Pulm Dis. (2017) 25(04):130–3.

[20] Nam H Jeon HE Kim WH Joa KL Lee H. Effect of maximal-intensity and high-intensity interval training on exercise capacity and quality of life in patients with acute myocardial infarction: a randomized controlled trial. Eur J Phys Rehabil Med. (2024) 60(1):104–12. 10.23736/s1973-9087.23.08094-2 37906165 PMC10938035

[21] Trachsel LD David LP Gayda M Henri C Hayami D Thorin-Trescases N The impact of high-intensity interval training on ventricular remodeling in patients with a recent acute myocardial infarction-A randomized training intervention pilot study. Clin Cardiol. (2019) 42(12):1222–31. 10.1002/clc.23277 31599994 PMC6906981

[22] Yakut H Dursun H Felekoğlu E Başkurt AA Alpaydın A Özalevli S. Effect of home-based high-intensity interval training versus moderate-intensity continuous training in patients with myocardial infarction: a randomized controlled trial. Ir J Med Sci. (2022) 191(6):2539–48. 10.1007/s11845-021-02867-x 34993836 PMC8736320

[23] Yi H Xin J Peng H Yang P. Effects of high-intensity interval training on cardiac rehabilitation in patients with acute myocardial infarction following percutaneous coronary intervention. J Xinxiang Med Univ. (2021) 38(7):645–8. 10.7683/xxyxyxb.2021.07.009

[24] Gao C Yue Y Wu D Zhang J Zhu S. Effects of high-intensity interval training versus moderate-intensity continuous training on cardiorespiratory and exercise capacity in patients with coronary artery disease: a systematic review and meta-analysis. PLoS One. (2025) 20(2):e0314134. 10.1371/journal.pone.0314134 39977401 PMC11841918

[25] Chen X Zhang T Hu X Wen Z Lu W Jiang W. High-intensity interval training programs versus moderate-intensity continuous training for individuals with heart failure: a systematic review and meta-analysis. Arch Phys Med Rehabil. (2025) 106(1):98–112. 10.1016/j.apmr.2024.05.028 38862032

[26] Gomes Neto M Durães AR Conceição LSR Saquetto MB Ellingsen Ø Carvalho VO. High intensity interval training versus moderate intensity continuous training on exercise capacity and quality of life in patients with heart failure with reduced ejection fraction: a systematic review and meta-analysis. Int J Cardiol. (2018) 261:134–41. 10.1016/j.ijcard.2018.02.076 29572084

[27] Košuta D Novaković M Božič Mijovski M Jug B. Acute effects of high intensity interval training versus moderate intensity continuous training on haemostasis in patients with coronary artery disease. Sci Rep. (2024) 14(1):1963. 10.1038/s41598-024-52521-6 38263210 PMC10806221

[28] Hackney KJ Bradley AP Roehl AS McGrath R Smith J. Energy expenditure and substrate utilization with hands-free crutches compared to conventional lower-extremity injury mobility devices. Foot Ankle Orthop. (2022) 7(4):24730114221139800. 10.1177/24730114221139800 36506649 PMC9729997

[29] Aispuru-Lanche R Jayo-Montoya JA Maldonado-Martín S. Vascular-endothelial adaptations following low and high volumes of high-intensity interval training in patients after myocardial infarction. Ther Adv Cardiovasc Dis. (2024) 18:17539447241286036. 10.1177/17539447241286036 39380195 PMC11483797

[30] Wang B Zhou R Wang Y Liu X Shou X Yang Y Effect of high-intensity interval training on cardiac structure and function in rats with acute myocardial infarct. Biomed Pharmacother. (2020) 131:110690. 10.1016/j.biopha.2020.110690 32890969

[31] Lund JS Aksetøy IA Dalen H Amundsen BH Støylen A. Left ventricular diastolic function: effects of high-intensity exercise after acute myocardial infarction. Echocardiography. (2020) 37(6):858–66. 10.1111/echo.14750 32497332

[32] Christou GA Christou MA Davos CH Markozannes G Christou KA Mantzoukas S Ergophysiological evaluation of heart failure patients with reduced ejection fraction undergoing exercise-based cardiac rehabilitation: a systematic review and meta-analysis. Hellenic J Cardiol. (2024) 77:106–19. 10.1016/j.hjc.2024.01.004 38246276

[33] Chen YW Wang CY Lai YH Liao YC Wen YK Chang ST Home-based cardiac rehabilitation improves quality of life, aerobic capacity, and readmission rates in patients with chronic heart failure. Medicine (Baltimore). (2018) 97(4):e9629. 10.1097/md.0000000000009629 29369178 PMC5794362

[34] Gaskill SE Skinner JS Quindry J. Ventilatory threshold related to v˙O 2 reserve, heart rate reserve, and rating of perceived exertion in a large varied sample. Med Sci Sports Exerc. (2023) 55(10):1876–85. 10.1249/MSS.0000000000003220 37202881 PMC10524184

[35] Ghanimati R Rajabi H Ramezani F Ramez M Bapiran M Nasirinezhad F. The effect of preconditioning with high-intensity training on tissue levels of G-CSF, its receptor and C-kit after an acute myocardial infarction in male rats. BMC Cardiovasc Disord. (2020) 20(1):75. 10.1186/s12872-020-01380-w 32046645 PMC7011373

[36] Lindgren M Börjesson M. The importance of physical activity and cardiorespiratory fitness for patients with heart failure. Diabetes Res Clin Pract. (2021) 176:108833. 10.1016/j.diabres.2021.108833 33895194

[37] Castagnoli R Pala F Subramanian P Oguz C Schwarz B Lim AI Immunopathological and microbial signatures of inflammatory bowel disease in partial RAG deficiency. J Exp Med. (2025) 222(8):e20241993. 10.1084/jem.20241993 40314722 PMC12047384

