# Supplementary material for: Effects of high-intensity interval training on cardiopulmonary function and quality of life in patients with myocardial infarction: a meta-analysis
Source: Front Cardiovasc Med. 2025 Dec 15;12:1666325. doi: 10.3389/fcvm.2025.1666325 (PMC12745437; doi:10.3389/fcvm.2025.1666325)
Supplement: Supplementary file 1 [file Table1.docx]

Supplementary Table S1

| Database | Retrieval strategy |
| --- | --- |
| Pubmed | ("Myocardial Infarction"[Mesh] OR "heart attack" OR "myocardial infarction" OR "acute myocardial infarction" OR "AMI") AND ("High-Intensity Interval Training"[Mesh] OR "high intensity interval training" OR "HIIT" OR "interval exercise") |
| Embase | ('myocardial infarction'/exp OR 'myocardial infarction' OR 'heart attack' OR 'AMI') AND ('high intensity interval training'/exp OR 'HIIT' OR 'interval exercise' OR 'interval training') |
| Web of Science | TS=("myocardial infarction" OR "heart attack" OR "AMI") AND TS=("high intensity interval training" OR "HIIT" OR "interval exercise") |
| Cochrane library | ([mh "Myocardial Infarction"] OR "myocardial infarction" OR "heart attack" OR "AMI") AND ([mh "High-Intensity Interval Training"] OR "high intensity interval training" OR "HIIT" OR "interval exercise") |
| CNKI | SU = ("High-Intensity Interval Training" OR "High-Intensity Intermittent Training" OR "HIIT") AND SU = ("Myocardial Infarction" OR "Acute Myocardial Infarction" OR "AMI") |
